# Fine motor skills at preschool age and later academic skills: an indirect-effects analysis involving executive function in high-altitude Tibetan preschoolers

**DOI:** 10.3389/fpsyg.2026.1938612

**Published:** 2026-09-08

**Authors:** Yibei Wang, Rui Su, Sonam Wangdue, Hejie Zhang, Jiaxin Chen, Chunmiao Wang, Yanfeng Zhang

**Affiliations:** 1 China Institute of Sport Science, Beijing, China; 2 Plateau Brain Science Research Center, Xizang University, Lhasa, China; 3 Xizang Autonomous Region Experimental Kindergarten, Lhasa, China

**Keywords:** early academic skills, executive function, fine motor skills, high altitude, preschoolers

## Abstract

Fine motor skills are associated with later academic development, but the role of executive function in this association remains insufficiently understood, particularly in high-altitude preschool populations. This two-wave half-longitudinal study examined the statistical indirect association between early fine motor skills and subsequent early academic skills involving executive function among high-altitude Tibetan preschoolers. The primary analytic cohort included 202 children born and raised in Lhasa, China (approximately 3,650 m). Fine motor skills, executive function, and early academic skills were assessed at baseline, with executive function and early academic skills reassessed approximately 10 months later. The primary structural equation model, adjusting for T2 child age, sex, T2 nonverbal intelligence, and family socioeconomic status, examined baseline fine motor skills in relation to follow-up executive function and early academic skills, with the latter specified as a latent factor indicated by mathematics, receptive vocabulary, and expressive vocabulary. T1 fine motor skills were positively associated with T2 executive function (β = 0.121, *p* = 0.016), and T2 executive function was positively associated with T2 early academic skills (β = 0.439, *p* < 0.001). The direct association between T1 fine motor skills and T2 early academic skills was not statistically significant (β = 0.020, *p* = 0.666). The statistical indirect association involving T2 executive function was small but statistically significant (*B* = 0.148, 95% CI [0.029, 0.281]; standardized β = 0.053), whereas the total association was not statistically significant (β = 0.073, *p* = 0.155). Because executive function and early academic skills were assessed concurrently at T2, this indirect association should not be interpreted as evidence of longitudinal or causal mediation. Rather, executive function may represent one potential statistical pathway involved in the observed association between earlier fine motor skills and subsequent early academic skills in this high-altitude preschool population.

## 1 Introduction

Motor skills develop rapidly from infancy through early childhood (Goodway et al., 2019). Through movement, exploration, and object manipulation, children acquire opportunities for learning that are closely linked to later cognitive and academic development (Diamond, 2000; Adolph, 2008). Among different domains of motor development, fine motor skills may be especially relevant to school readiness because they require precise hand and finger movements coordinated with perception, attention, and goal-directed control, and are frequently involved in classroom activities such as drawing, tracing, manipulating learning materials, and early writing (Marr et al., 2003; Cameron et al., 2012). Fine motor skills constitute a broad domain that includes multiple aspects of precise hand-finger control, among which manual dexterity and visuomotor integration are related but distinguishable constructs. Recent meta-analytic evidence supports a significant positive association between preschool children's fine motor skills and academic achievement, particularly mathematics; however, most existing studies remain correlational, highlighting the need to examine potential pathways underlying the association between fine motor skills and later academic development (Li et al., 2024).

Like fine motor skills, executive function (EF) has been conceptualized as a foundational skill for school readiness because it reflects the underlying cognitive processes that allow children to engage effectively in learning activities rather than the academic content already acquired (McClelland et al., 2007; Spiegel et al., 2021; Turnbull et al., 2024). In developmental research, EF is commonly defined as a set of higher-order cognitive control processes that support purposeful, goal-directed, and future-oriented behavior, with core components including inhibitory control, working memory, and cognitive flexibility (Diamond, 2013; Karr et al., 2018). Evidence from preschool samples suggests substantial shared variance across these EF domains. Wiebe et al. (2008) found that a single general executive-control factor adequately represented performance across multiple EF tasks in preschool children. Consistent with this perspective, previous research with children assessed at ages 3, 4, and 5 has also summarized performance across multiple EF tasks using an overall composite score (Willoughby et al., 2017). Importantly, EF may be involved in the association between early fine motor skills and later academic skills. From an embodied-cognition perspective, cognitive processes emerge through ongoing interactions among perception, action, and environmental demands (Thelen et al., 2001; Wilson, 2002). Fine motor activities are therefore not purely motoric; successful performance requires children to coordinate perception and action while maintaining task goals, selecting and sequencing actions, inhibiting competing responses, updating task-relevant information, and monitoring ongoing performance (Grissmer et al., 2010). These demands overlap with core executive-control processes, providing a theoretical basis for expecting fine motor performance and executive function to be related during goal-directed activity. Executive processes are also involved in early mathematics and language learning, where children must maintain relevant information, inhibit competing responses, flexibly shift between representations, and organize goal-directed responses. For example, mathematics learning requires children to hold and manipulate quantitative information, shift between symbols and meanings, and inhibit incorrect strategies (Viterbori et al., 2015; Hawes et al., 2019), whereas language learning requires children to maintain attention to verbal input, update lexical and semantic information, and organize responses during comprehension and expression (Connor et al., 2016; Cirino et al., 2019). Thus, EF may be associated with later academic skills across multiple domains.

Embodied cognition therefore provides a conceptual rationale for examining EF as a potential intermediate variable in the association between earlier fine motor skills and subsequent early academic skills, rather than implying that fine motor skills causally strengthen EF or that EF constitutes a demonstrated developmental mediator. Consistent with this perspective, developmental research suggests that motor and cognitive processes are closely intertwined (McClelland and Cameron, 2019), and Schmidt et al. (2017) reported that EF mediated the association between children's motor ability and later academic achievement. Recent preschool research has also situated motor and executive-function development within broader movement and ecological contexts. Intervention studies have examined whether structured movement activities influence motor and executive-function outcomes (Ltifi et al., 2025a), whereas observational work has examined motor skills and executive functions alongside 24-h movement behaviors (Ltifi et al., 2025b, 2026b; Meksi et al., 2026). Together, this literature places motor and executive processes within broader behavioral contexts without establishing causal directionality.

It is estimated that more than 140 million people live at altitudes above 2,500 m worldwide (Penaloza and Arias-Stella, 2007). High-altitude environments therefore provide an important ecological context for developmental research, especially among preschool children, for whom motor skills, EF, and early academic abilities are undergoing rapid and interconnected development. As a developmental context, high altitude is characterized by distinctive ecological and physiological conditions (Niermeyer et al., 2009; Hogan et al., 2010). Recent preschool research has also reported urban-rural differences in movement behaviors, motor skills, and executive functions, underscoring the relevance of broader residential context (Ltifi et al., 2026a). However, existing high-altitude research has focused primarily on school-aged children or adults (Liu et al., 2023; Su et al., 2024), leaving preschool children under-represented in studies of early motor, cognitive, and academic development. Moreover, whether early fine motor skills are associated with executive function and early academic skills later in preschool children living in high-altitude contexts remains to be further explored.

The present study therefore examined a two-wave half-longitudinal indirect-effects model among Tibetan preschoolers living at approximately 3,650 m in Lhasa, China. Early academic skills in preschool encompass multiple related domains of emerging competence, particularly early numeracy and language skills, both of which are central to children's readiness for formal learning (Duncan et al., 2007; McClelland et al., 2007). Although mathematics and language represent conceptually distinct domains, substantial evidence indicates that they are developmentally interrelated. A large meta-analysis, for example, found a moderate association between language and mathematics across development and further showed that the two domains longitudinally predicted one another after accounting for initial performance (Peng et al., 2020). Accordingly, in the present study, mathematics, receptive vocabulary, and expressive vocabulary were specified as separate observed indicators of a broader latent early-academic-skills construct. The latent factor was intended to capture the shared variance across these quantitative and language-related domains of early learning, rather than to imply that mathematics and vocabulary are interchangeable or constitute a single homogeneous ability. Fine motor skills, EF, and early academic skills were assessed at baseline, with EF and early academic skills reassessed approximately 10 months later. The primary model examined whether baseline fine motor skills were associated with follow-up EF and whether there was a statistical indirect association between baseline fine motor skills and follow-up early academic skills involving follow-up EF. We expected earlier fine motor skills to be positively associated with follow-up EF, and follow-up EF to be positively associated with early academic skills. Baseline EF and early academic measures were additionally included in a supplementary baseline-adjusted sensitivity analysis. Because EF and early academic skills were assessed concurrently at follow-up, temporal ordering was established only from baseline fine motor skills to the follow-up measures. Accordingly, the model was interpreted as a half-longitudinal indirect-effects model rather than as a test of longitudinal or causal mediation.

## 2 Methods

### 2.1 Participants

**Figure 1:**
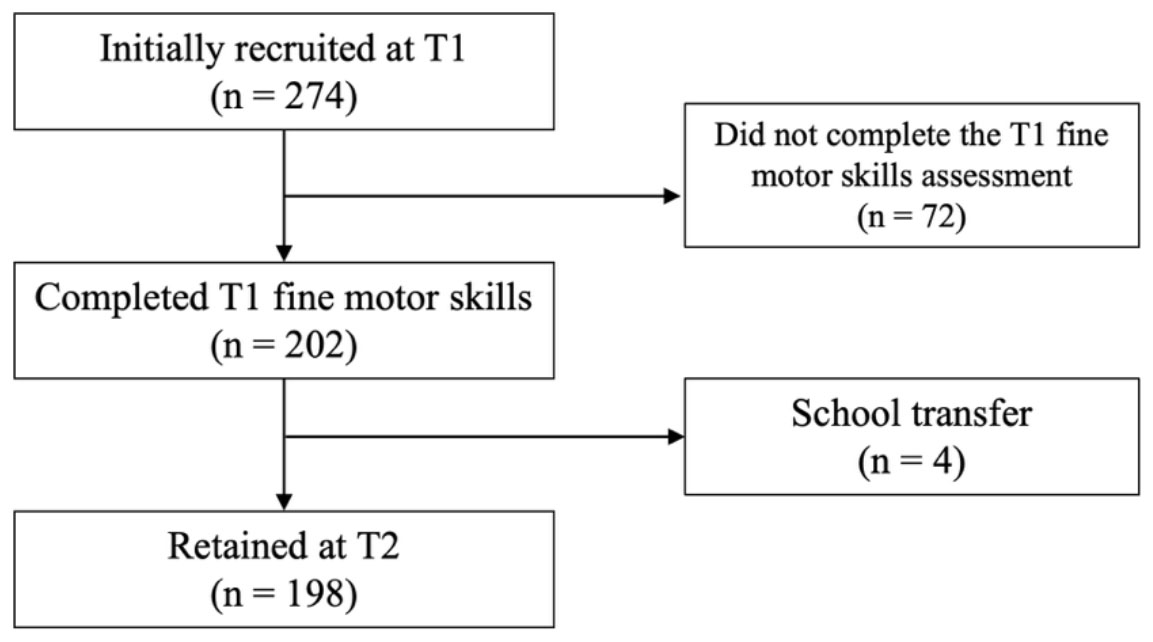
Participant flow from T1 recruitment to T2 follow-up.

Preschool children enrolled in a single kindergarten in Lhasa, Xizang Autonomous Region, China, located at an elevation of approximately 3,650 m, were invited to participate through the kindergarten. Participation required written informed consent from a parent or legal guardian, and eligibility was restricted to children of Tibetan ethnicity at recruitment. All participating children were born and raised in Lhasa and had lived at high altitude since birth. Children were excluded if parental reports or existing preschool records indicated a previous diagnosis of intellectual disability, autism spectrum disorder, attention-deficit/hyperactivity disorder, or learning disorder. A total of 274 children were initially recruited at T1. Because an observed T1 fine motor skills score was required for inclusion in the primary analysis, 202 children who completed the T1 fine motor skills assessment constituted the primary analytic cohort, whereas 72 children who did not complete this assessment were not included in the primary analytic cohort. Among the 202 children in the primary analytic cohort, 198 participated in the T2 assessment, whereas four were lost to follow-up because of school transfer, corresponding to a retention rate of 98.0%. The primary analytic cohort comprised 102 boys (50.5%) and 100 girls (49.5%). Missing data on other study variables within the primary analytic cohort were addressed using multiple imputation. Participant flow is summarized in Figure 1. Descriptive T1 characteristics of participants retained and lost to follow-up are presented in Supplementary Table S1.

In the primary analytic cohort, mean age at baseline was 48.71 months (*SD* = 6.83; range = 36.86–61.61). Among the 198 children reassessed at follow-up, mean age was 59.50 months (*SD* = 6.71; range = 47.01–72.33). The mean assessment interval was 10.30 months (*SD* = 0.49; range = 8.97–11.53). Ethical approval was granted by the Medical Ethics Review Committee of Xizang University (Approval No. ZDYXLL2025002). Written informed consent was obtained from all parents or legal guardians prior to data collection.

### 2.2 Measures

#### 2.2.1 Fine motor skills

Fine motor skills were assessed using the Manual Dexterity component of the standardized Movement Assessment Battery for Children-Second Edition (MABC-2) (Hua et al., 2013). Previous research examining MABC-2 Age Band 1 in mainland Chinese children has reported acceptable reliability and validity, including evidence of internal consistency, test-retest and inter-rater reliability, and factorial validity (Hua et al., 2013). The Manual Dexterity tasks require precise and coordinated hand and finger movements, including placing pegs, threading, and drawing a trail. Raw scores were converted to standard scores ranging from 1 to 19, with higher scores indicating better manual-dexterity performance.

#### 2.2.2 Executive function

Executive function (EF) was conceptualized as a broad construct encompassing three core components: inhibitory control, working memory, and cognitive flexibility. It was operationalized using five performance-based tasks, yielding four indicators: inhibitory control, auditory working memory, visual working memory, and cognitive flexibility.

Inhibitory control was measured with two tasks adapted from the Stroop paradigm: the Day-Night task and the Happy-Sad task (Gerstadt et al., 1994). In each task, children were required to give a response opposite to the prepotent one (e.g., saying “night” when shown a picture of the sun), thereby inhibiting an automatic response. Because both tasks assess Stroop-like inhibitory control using comparable rule-based response demands, their scores were combined to form the inhibitory-control indicator. Performance was similar across the Day-Night (*M* = 16.55, *SD* = 3.42) and Happy-Sad (*M* = 16.70, *SD* = 3.58) tasks. The two task scores were strongly correlated (*r* = 0.68), and the Spearman-Brown reliability coefficient for the combined indicator was 0.81. The mean number of correct responses across the two tasks was used as the index of inhibitory control, with higher scores indicating better inhibitory control.

Working memory comprised both auditory and visual components. Auditory working memory was assessed using a digit-span task in which children repeated sequences of digits in forward and backward order. The score was calculated as the sum of the longest correctly recalled forward and backward digit spans, with possible scores ranging from 0 to 18 (Chen and Stevenson, 1988). Visual working memory was assessed using a self-ordered pointing task that included six stimulus sets across a total of 12 trials, with scores ranging from 0 to 12 (Hongwanishkul et al., 2005).

Cognitive flexibility was assessed using the Dimensional Change Card Sort task from the Preschool Executive Function Toolbox (Howard and Melhuish, 2017). Children were required to sort cards according to one rule (e.g., color) and then switch to a different rule (e.g., shape), with performance on rule-switching trials reflecting the level of cognitive flexibility. Scores ranged from 0 to 12.

The four EF indicators—inhibitory control, auditory working memory, visual working memory, and cognitive flexibility—were z-standardized and averaged to form a multidomain EF composite, with higher scores indicating better overall EF performance. Internal consistency across the four standardized indicators was modest (standardized α = 0.48). This value was interpreted in the context of the multidimensional nature of EF assessment in early childhood, where performance-based EF tasks may show modest intercorrelations because they reflect both common executive processes and task-specific cognitive demands (Willoughby and Blair, 2016). The composite was used as the intermediate variable in the two-wave half-longitudinal indirect-effects model. This approach was based on the unity-and-diversity framework of executive function, which recognizes that inhibitory control, working memory, and cognitive flexibility represent related but partially distinct components that share common executive-control variance (Miyake et al., 2000; Friedman and Miyake, 2017). Therefore, the composite was intended to capture shared executive-function variance across these domains rather than to imply that they constitute an entirely unitary construct.

#### 2.2.3 Early academic skills

Early academic skills were indexed by early mathematics ability together with receptive and expressive language ability.

Early mathematics ability was assessed using the Test of Early Mathematics Ability-Third Edition (TEMA-3) (Ginsburg and Baroody, 2003), a standardized measure of children's early numerical and mathematical competence, including counting, number comparison, calculation, and basic number concepts.

Receptive vocabulary was assessed using the Peabody Picture Vocabulary Test-Revised (PPVT-R) (Lu and Liu, 1998), in which the examiner spoke a word and the child pointed to the corresponding picture among four options. Expressive vocabulary was assessed using the Expressive Vocabulary Test-Third Edition (EVT-3) (Williams, 2019), in which the child was shown a picture and asked to provide a one-word label or synonym. Both vocabulary measures are widely used standardized indices of children's language ability and school readiness.

The mathematics and vocabulary measures were administered in Mandarin versions. Mathematics, receptive vocabulary, and expressive vocabulary were modeled as separate observed indicators of a latent early-academic-skills factor representing their shared variance across quantitative and language-related domains of early learning.

#### 2.2.4 Covariates

Family socioeconomic status (SES) was indexed using parental education and family income. Maternal and paternal education were each assessed on an 8-point ordinal scale (1 = never attended school, 2 = literacy class, 3 = primary school, 4 = junior high school, 5 = senior high/vocational school, 6 = junior college, 7 = bachelor's degree, 8 = postgraduate degree or above). Family income was assessed using a 9-point ordinal scale of annual household income, ranging from 1 (below 10,000) to 9 (above 500,000). The two parental education scores and the family income score were standardized and averaged to create a composite SES index.

Nonverbal intelligence was assessed at both T1 and T2 using the Block Design and Picture Completion subtests of the China-Wechsler Younger Children Scale of Intelligence (C-WYCSI) (Gong and Dai, 1988). For the primary analyses, the T2 subtest scores were standardized and averaged to form the nonverbal-intelligence composite used as a covariate. Nonverbal-intelligence measures from both waves were included in the multiple-imputation model.

### 2.3 Procedure

Data were collected at baseline and approximately 10 months later. At both waves, all assessments were administered individually by trained research assistants in a quiet room at the kindergarten during regular school hours. Prior to data collection, the research assistants completed 1 week of standardized training in the administration and scoring of all study measures. Follow-up assessors were blinded to children's baseline performance. All assessments were administered in Mandarin Chinese. Mandarin Chinese was also the language of instruction at the participating kindergarten, and the children therefore had regular classroom exposure to Mandarin. When children had difficulty understanding task instructions, the accompanying classroom teacher clarified the procedural instructions as needed. Parents also reported the language primarily used by the child at home. Among the 202 children in the primary analytic cohort, 17 (8.4%) primarily used Mandarin, 43 (21.3%) primarily used Tibetan, and 142 (70.3%) used both Mandarin and Tibetan at home. Reported home language was treated as an indicator of children's linguistic background rather than as a direct measure of individual Mandarin proficiency.

Fine motor skills, EF, early academic skills, and nonverbal intelligence were assessed at baseline. EF, early academic skills, and nonverbal intelligence were reassessed at follow-up across three sessions to reduce fatigue and maintain children's engagement, with short breaks provided between tasks as needed.

Sociodemographic information, including child sex and date of birth, parental education, and family income, was collected through a questionnaire completed by the children's parents or guardians and returned to the kindergarten during the assessment period.

### 2.4 Statistical analysis

Missing data were present for several variables, primarily because some children were unavailable for particular assessment sessions and some parent-report questionnaires were incompletely returned. Across the variables included in the imputation model, 93 children (46.0%) had complete data and 109 (54.0%) had at least one missing value; variable-specific missingness is reported in Supplementary Table S2. Missing data were addressed using multiple imputation by chained equations under a missing-at-random assumption conditional on the variables included in the imputation model. A total of 50 imputed datasets were generated using the mice package in R (van Buuren and Groothuis-Oudshoorn, 2011). Numeric variables with missing values, including the ordinal socioeconomic indicators treated as numeric scores, were imputed using predictive mean matching. The imputation model included executive-function task scores, academic measures, and nonverbal-intelligence measures from both waves, together with family socioeconomic indicators, T2 age, sex, home language, and T1 fine motor skills. T1 fine motor skills were fully observed within the primary analytic cohort and were therefore not imputed. Parameter estimates were pooled using Rubin's rules. To evaluate potential selection bias associated with missing baseline fine motor assessments, baseline characteristics were compared between children included in the primary analytic cohort and those excluded due to missing T1 fine motor scores.

All analyses were conducted in R. Means, standard deviations, and ranges were computed for all study variables. Zero-order correlations were calculated to describe the bivariate associations among the study variables. Correlations were estimated within each of the 50 imputed datasets and pooled across imputations. Covariate-adjusted associations were evaluated in the primary SEM, which included T2 child age, sex, T2 nonverbal intelligence, and family socioeconomic status as covariates. As a supplementary psychometric analysis, a one-factor confirmatory factor analysis (CFA) was conducted to examine the common variance among the four EF indicators.

The primary hypothesis was tested using a two-wave half-longitudinal indirect-effects model estimated with structural equation modeling (SEM), following methodological considerations regarding longitudinal mediation analysis (Cole and Maxwell, 2003; Maxwell and Cole, 2007). The model was fitted to 50 imputed datasets using the MLR estimator, and parameter estimates were pooled according to Rubin's rules. Follow-up early academic skills were specified as a latent factor indicated by mathematics, receptive vocabulary, and expressive vocabulary. Baseline fine motor skills served as the predictor and the follow-up multidomain EF composite as the intermediate variable. The model estimated the baseline fine motor skills-follow-up EF association (path a), the concurrent association between follow-up EF and early academic skills (path b), and the direct association between baseline fine motor skills and follow-up early academic skills (path c'). T2 child age, sex, T2 nonverbal intelligence, and family socioeconomic status were included as covariates of both T2 EF and T2 early academic skills.

The statistical indirect association was estimated as the product of paths a and b, with its 95% confidence interval obtained using a Monte Carlo approach based on the Rubin-pooled parameter estimates and covariance matrix, following the general framework described by Pesigan and Cheung (2024). Because EF and early academic skills were assessed concurrently at follow-up, their temporal ordering could not be established. The indirect association was therefore interpreted statistically within a half-longitudinal model rather than as evidence of longitudinal or causal mediation. The primary model estimated follow-up levels rather than within-person change.

Because baseline EF and early academic measures were available, a supplementary baseline-adjusted sensitivity analysis was conducted. Follow-up EF was additionally adjusted for baseline EF, and follow-up early academic skills were adjusted for a baseline early-academic composite. Baseline EF was constructed using the same standardized four-indicator approach as the follow-up EF measure, whereas the baseline early-academic composite was calculated by z-standardizing and averaging mathematics, receptive-vocabulary, and expressive-vocabulary scores. This analysis examined whether the primary statistical indirect association remained evident after accounting for corresponding baseline levels; it was not interpreted as a model of within-person developmental change. As an additional sensitivity analysis related to longitudinal attrition and missing-data handling, the primary model was re-estimated among the 198 children who participated at T2, and the resulting estimates were compared with those from the primary *N* = 202 analysis. Given the linguistic diversity of the sample, an additional sensitivity analysis was conducted in which reported home language was included as a covariate of both T2 executive function and T2 early academic skills. Home language was represented by two dummy-coded indicators for Tibetan and bilingual Mandarin-Tibetan use, with Mandarin-only home language serving as the reference category.

Model fit was evaluated using the chi-square statistic (χ^2^), comparative fit index (CFI), Tucker-Lewis index (TLI), root mean square error of approximation (RMSEA), and standardized root mean square residual (SRMR). The reporting of this observational longitudinal study was guided by the STROBE recommendations.

## 3 Results

### 3.1 Participant characteristics

Descriptive statistics for the primary study variables are presented in Table 1. Comparisons between children included in the primary analytic cohort (*n* = 202) and those excluded due to missing T1 fine motor assessments (*n* = 72) showed no significant differences in sex, home language, parental education, or family income. Excluded children were younger than included children (46.18 vs. 48.71 months, *p* = 0.004). Among available baseline executive-function indicators, excluded children showed lower digit span performance (*p* = 0.028) but higher self-ordered pointing performance (*p* = 0.008), whereas other indicators did not differ significantly between groups.

**Table 1:** Descriptive statistics for primary study variables.

Variable	M	SD	Min	Max
Age at T1, months	48.71	6.83	36.86	61.61
Age at T2, months	59.50	6.71	47.01	72.33
Fine motor skills	9.26	2.75	1.00	18.00
Executive function				
Inhibition	16.62	3.21	3.50	20.00
Auditory WM	5.60	1.65	2.00	10.00
Visual WM	7.51	2.97	2.00	12.00
Cognitive flexibility	5.82	4.02	0.00	12.00
Early academic skills				
Mathematics	22.07	9.72	3.00	46.00
Receptive vocab	44.73	22.24	13.00	99.00
Expressive vocab	58.57	15.36	24.00	101.00
Nonverbal intelligence	22.43	9.14	5.00	45.00
Family socioeconomic status				
Paternal education	6.33	1.35	1.00	8.00
Maternal education	6.31	1.46	1.00	8.00
Family annual income	6.16	2.32	1.00	9.00

Fine motor skills are reported at T1; executive-function, early-academic-skills, and nonverbal-intelligence values correspond to T2 measures used in the primary model.

### 3.2 Zero-order correlations

Zero-order correlations among the study variables are presented in Table 2. T1 fine motor skills showed positive associations with T2 auditory working memory (*r* = 0.23, *p* < 0.01), mathematics (*r* = 0.19, *p* < 0.01), receptive vocabulary (*r* = 0.24, *p* < 0.001), and nonverbal intelligence (*r* = 0.27, *p* < 0.001). T2 executive-function indicators were also positively associated with early academic skills. For example, auditory working memory was associated with mathematics (*r* = 0.56, *p* < 0.001), receptive vocabulary (*r* = 0.45, *p* < 0.001), and expressive vocabulary (*r* = 0.46, *p* < 0.001), while cognitive flexibility was associated with mathematics (*r* = 0.43, *p* < 0.001), receptive vocabulary (*r* = 0.42, *p* < 0.001), and expressive vocabulary (*r* = 0.36, *p* < 0.001). The early-academic-skills indicators were also positively intercorrelated, including mathematics with receptive vocabulary (*r* = 0.58, *p* < 0.001), mathematics with expressive vocabulary (*r* = 0.53, *p* < 0.001), and receptive with expressive vocabulary (*r* = 0.66, *p* < 0.001). These zero-order correlations are presented as descriptive bivariate associations; covariate-adjusted associations were evaluated in the primary SEM.

**Table 2:** Zero-order correlations among study variables.

Variable	1	2	3	4	5	6	7	8	9	10
1. Fine motor skills	—									
2. Inhibition	0.14	—								
3. Auditory WM	0.23^**^	0.24^***^	—							
4. Visual WM	0.13	0.14^*^	0.15^*^	—						
5. Cognitive flexibility	0.12	0.20^**^	0.26^***^	0.13	—					
6. Mathematics	0.19^**^	0.42^***^	0.56^***^	0.21^**^	0.43^***^	—				
7. Receptive vocabulary	0.24^***^	0.42^***^	0.45^***^	0.36^***^	0.42^***^	0.58^***^	—			
8. Expressive vocabulary	0.14	0.40^***^	0.46^***^	0.23^**^	0.36^***^	0.53^***^	0.66^***^	—		
9. Nonverbal intelligence	0.27^***^	0.34^***^	0.46^***^	0.16^*^	0.41^***^	0.62^***^	0.62^***^	0.54^***^	—	
10. Socioeconomic status	0.06	−0.01	0.26^***^	0.13	0.23^**^	0.20^*^	0.22^**^	0.27^***^	0.23^**^	—

Fine motor skills values shown in the table are from T1, whereas the executive-function, early-academic-skills, and nonverbal-intelligence values shown are from T2. ^***^p < 0.001; ^**^p < 0.01; ^*^p < 0.05.

### 3.3 Indirect-effects analysis using structural equation modeling

Before testing the indirect-effects model, a supplementary one-factor CFA was conducted as a psychometric check of the common variance among the four EF indicators. All four indicators loaded significantly on the common EF factor (standardized loadings = 0.29–0.55, all *p* < 0.01). Because the model had only two degrees of freedom, global fit indices were not overinterpreted. These results indicated shared variance across the EF indicators while also suggesting heterogeneity among the individual components. The primary half-longitudinal indirect-effects model specified T2 early academic skills as a latent factor indicated by mathematics, receptive vocabulary, and expressive vocabulary. The three indicators loaded positively on the latent factor (standardized loadings = 0.72–0.80, all *ps* < 0.001). The model showed acceptable overall fit, χ^2^ (12) = 23.31, *p* = 0.025, CFI = 0.977, TLI = 0.950, RMSEA = 0.068, and SRMR = 0.028.

**Figure 2:**
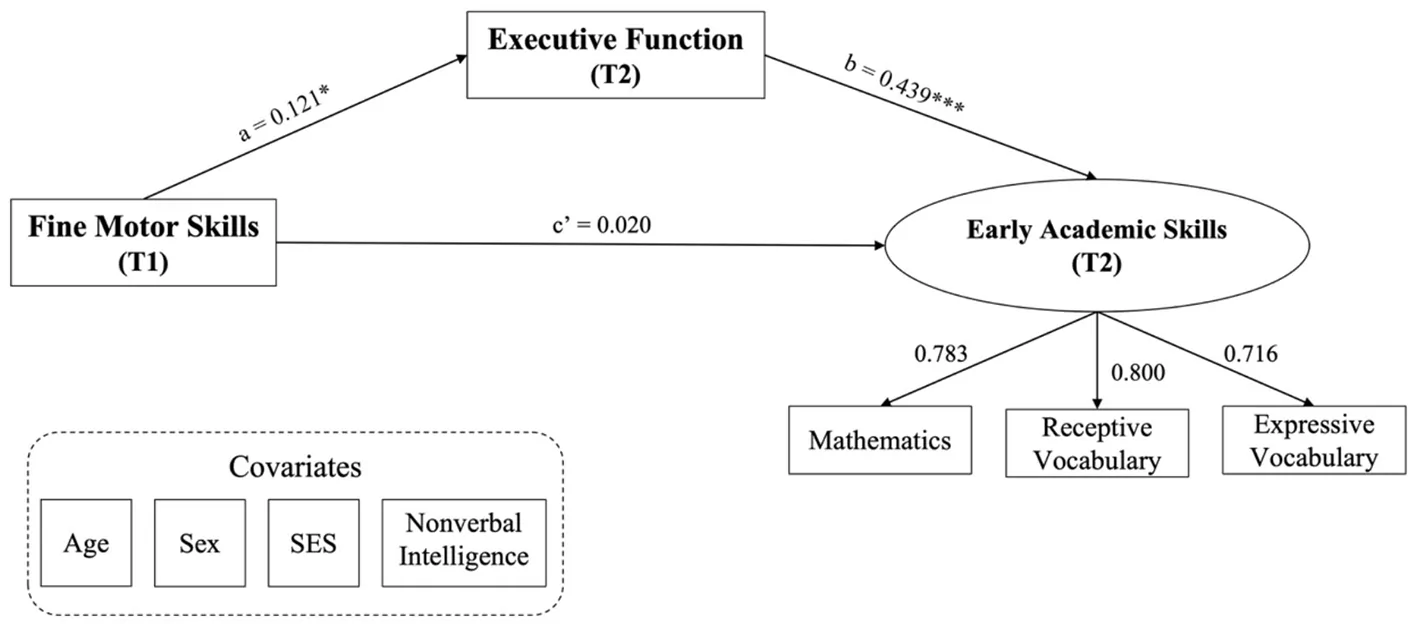
Primary two-wave half-longitudinal indirect-effects model. Values are standardized estimates. Covariate paths from age, sex, family socioeconomic status, and nonverbal intelligence to both T2 executive function and T2 early academic skills were estimated but omitted for visual clarity. *p < 0.05; ***p < 0.001.

Standardized path estimates are reported in Table 3 and depicted in Figure 2. T1 fine motor skills were positively associated with T2 executive function (β = 0.121, *p* = 0.016), and T2 executive function was positively associated with T2 early academic skills (β = 0.439, *p* < 0.001). The direct association between T1 fine motor skills and T2 early academic skills was not statistically significant (β = 0.020, *p* = 0.666). The statistical indirect association involving T2 executive function was small but statistically significant (*B* = 0.148, 95% CI [0.029, 0.281]; standardized β = 0.053), whereas the total association was not statistically significant (β = 0.073, *p* = 0.155). All structural paths were adjusted for child age, sex, nonverbal intelligence, and family socioeconomic status.

**Table 3:** Statistical indirect association between T1 fine motor skills and T2 early academic skills involving T2 executive function.

Path/parameter	*B*	SE	β	*p*	95% CI
Structural paths					
a: Fine motor skills → EF	0.028	0.011	0.121	0.016	[0.006, 0.050]
b: EF → Early academic skills	5.357	0.752	0.439	<0.001	[3.883, 6.831]
c′: Fine motor skills → Early academic skills	0.055	0.127	0.020	0.666	[−0.194, 0.304]
Effect decomposition					
Indirect association (a × b)	0.148	–	0.053	–	[0.029, 0.281]
Total association	0.203	0.143	0.073	0.155	[−0.077, 0.483]

EF, executive function. All structural paths were adjusted for T2 child age, sex, T2 nonverbal intelligence, and family socioeconomic status. Monte Carlo 95% CI is reported for the statistical indirect association.

In a supplementary baseline-adjusted sensitivity analysis, T1 fine motor skills remained positively associated with T2 executive function after adjustment for T1 executive function (β = 0.114, *p* = 0.028), and T2 executive function remained positively associated with T2 early academic skills after adjustment for the T1 early-academic composite (β = 0.250, *p* < 0.001). The statistical indirect association was attenuated but remained statistically significant (*B* = 0.076, 95% CI [0.010, 0.160]; standardized β = 0.029). In a separate sensitivity analysis restricted to the 198 children who participated at T2, the standardized indirect association was very similar to that obtained in the full N = 202 analysis (β = 0.052 vs. β = 0.053), suggesting that the primary estimate was not materially altered by longitudinal attrition. In an additional sensitivity analysis adjusting for reported home language, the standardized indirect association also remained very similar to that observed in the primary model (β = 0.050 vs. β = 0.053), indicating that the primary estimate was not materially altered after adjustment for home-language background.

## 4 Discussion

Using a two-wave half-longitudinal indirect-effects model and adjusting for child age, sex, nonverbal intelligence, and family socioeconomic status, we found a statistically significant indirect association between T1 fine motor skills and T2 early academic skills involving T2 executive function.

### 4.1 Fine motor skills and early academic development

Fine motor skills represent a foundational domain of early childhood development, reflecting children's ability to coordinate visually guided goal-directed actions. Beyond motor coordination, fine motor skills are increasingly recognized as early indicators of school readiness and broader neurocognitive development (Cameron et al., 2012; Needham and Nelson, 2023). The academic relevance of fine motor skills may depend more on their cognitively demanding components than on manual dexterity alone. Carlson et al. (2013) found that visual-spatial integration, rather than visual-motor coordination, uniquely predicted children's mathematics and written expression achievement after controlling for gender, socioeconomic status, IQ, and visual-motor coordination. Similarly, Kim et al. (2018) showed that fine motor coordination contributed to children's mathematics development indirectly through visuomotor integration across kindergarten and first grade. Research distinguishing different components of fine motor skills has shown that fine motor skills are stronger predictors of early mathematics than of early reading (Pitchford et al., 2016). Moreover, longitudinal evidence suggests that fine motor skills and executive functions are jointly associated with later mathematics-related outcomes. Fine motor skills, executive functions, and basic numerical skills in kindergarten have each been shown to make unique contributions to later mathematics achievement (Gashaj et al., 2019). Similarly, kindergarten fine motor skills have been found to predict arithmetic skills in first and second grade, and when executive functions were considered, visuospatial working memory and interference control emerged as important predictors (Michel et al., 2020). Together, these findings suggest that fine motor tasks may be linked to academic learning because they require children to integrate visual information, spatial organization, motor planning, and ongoing performance monitoring—processes that overlap substantially with executive function.

### 4.2 Executive function in the link between fine motor skills and early academic skills

The observed pattern is consistent with executive function being statistically involved in the association between earlier fine motor skills and subsequent early academic skills, but it should be interpreted cautiously because the indirect association was small, the total association was not statistically significant, and EF and early academic skills were assessed concurrently at T2. Furthermore, the baseline-adjusted sensitivity analysis showed an attenuated but still statistically significant indirect association, suggesting that part of the observed association may reflect individual differences already present at baseline. Therefore, the present findings do not establish a temporal mediating role of executive function but indicate a statistical indirect association involving executive function that requires further evaluation using designs with temporally separated assessments. This pattern can also be considered in relation to developmental cascade theory, which provides a theoretical framework for understanding how developmental characteristics may be associated across domains over time. However, because the present observational design does not establish temporal ordering among EF and early academic skills, the findings should be interpreted as consistent with, rather than a direct test of, a developmental cascade (McClelland and Cameron, 2019; Willoughby and Hudson, 2023; Zhou and Tolmie, 2024). Prior work has proposed partially overlapping neural systems for motor and executive control (Diamond, 2000), but no neurobiological variables were measured in the present study. At the behavioral level, motor tasks may recruit executive-control processes, suggesting that motor and executive processes can be jointly engaged during goal-directed activity (Bao et al., 2024; Hill et al., 2024).

Embodied cognition emphasizes that cognitive processes emerge through continuous interactions among perception, action, and environmental constraints (Thelen et al., 2001; Wilson, 2002). From this perspective, fine motor activities are not merely motoric acts; rather, the perception-action coordination they require places demands on cognitive-control processes such as goal maintenance, action planning, response inhibition, updating, and performance monitoring. These demands overlap with core components of executive function, providing a theoretical basis for examining the statistical association between earlier fine motor skills and executive-function performance. Importantly, many of these same executive processes are also involved in early academic learning. In mathematics, children must maintain and manipulate task-relevant information, inhibit competing responses, and flexibly shift between representations, whereas language learning similarly requires the maintenance and updating of verbal information, inhibition of irrelevant responses, and flexible selection of appropriate meanings or responses. Within this framework, executive function may therefore represent one potential statistical pathway involved in the association between earlier fine motor skills and subsequent early academic skills. This interpretation is consistent with evidence that executive function is closely related to core domains of early academic learning. For mathematics, meta-analytic evidence in preschool children indicates that executive functions are positively associated with mathematical ability, with inhibition, shifting, and updating all showing significant relations to early math performance (Emslander and Scherer, 2022). For language, executive functions have been linked to processes involved in maintaining communicative goals, updating verbal information, inhibiting irrelevant responses, and flexibly selecting appropriate words or meanings. Consistent with this view, recent review evidence suggests that executive functions and language skills are closely linked during childhood, although their developmental directionality may be complex and potentially bidirectional (Shokrkon and Nicoladis, 2022). Taken together, this literature provides a theoretical basis for examining EF as a potential statistical pathway. Embodied cognition is used here as an interpretive framework rather than as a mechanism tested by the present data.

### 4.3 High-altitude developmental context

An important contextual contribution of this study is its focus on Tibetan preschool children living at approximately 3,650 m, representing an underrepresented population in research on motor, cognitive, and academic development. In the present study, high altitude was treated as a developmental and ecological context rather than as a causal explanatory factor. We did not measure oxygen saturation or other physiological indicators of hypoxia or adaptation, nor did we compare children across different altitudes. Accordingly, the present analyses characterize associations among fine motor skills, executive function, and early academic skills within this high-altitude population rather than effects attributable to altitude itself. The statistical indirect association remained evident after adjustment for the measured SES indicators, although unmeasured family and contextual factors may still have contributed to the observed pattern. Comparative studies across altitude levels, ideally incorporating direct physiological measures, are needed to determine whether altitude modifies the magnitude or structure of these associations.

### 4.4 Limitations

Several limitations should be considered when interpreting these findings. First, EF and early academic skills were assessed concurrently at T2, so temporal precedence between these constructs cannot be established. Although baseline measures were included in a sensitivity analysis, the primary model estimated T2 levels rather than within-person change; studies with three or more waves are needed to test temporal pathways more rigorously. Second, participants were recruited from a single kindergarten in Lhasa, which may limit the generalizability of the findings. Third, although several key covariates were included, residual confounding remains possible because unmeasured factors, such as the home cognitive environment, educational experiences, physical activity, nutrition, and individual language proficiency, may have influenced the observed associations among fine motor skills, executive function, and early academic skills. Fourth, although Mandarin was the language of kindergarten instruction and all children had regular classroom exposure to Mandarin, individual Mandarin proficiency was not formally assessed. Reported home language may not fully capture individual differences in Mandarin comprehension or expressive proficiency, which could have influenced performance on the language-based measures. In addition, the cultural validity of the measures has not been specifically established in Tibetan preschoolers. Moreover, the multidomain EF composite showed modest internal consistency and heterogeneous contributions across indicators, and findings involving this composite should therefore be interpreted cautiously. In the present study, EF was conceptualized as a multidomain construct with related but distinguishable components, and the composite served as an operational summary of inhibitory control, auditory and visual working memory, and cognitive flexibility rather than as a strictly unidimensional scale. This approach was consistent with the unity-and-diversity framework of EF, which recognizes shared executive-control variance across partially distinct components. Although the composite approach was supported by the unity-and-diversity framework of EF, inhibitory control, working memory, and cognitive flexibility represent partially distinct domains that may follow different developmental trajectories and contribute differently to academic development. Future studies should examine domain-specific EF pathways using repeated assessments of individual EF components. In addition, fine motor skills were assessed using the Manual Dexterity component of the Movement Assessment Battery for Children-Second Edition (MABC-2). Therefore, the findings primarily reflect a manual-dexterity-based aspect of fine motor skills rather than the entire fine-motor domain or visuomotor integration specifically. Finally, no direct physiological indicators of altitude or hypoxia were included, limiting conclusions about the specific role of the high-altitude environment. Future research incorporating more diverse samples, repeated assessments, and physiological measures is warranted.

## 5 Conclusion

This two-wave half-longitudinal study identified a small but statistically significant indirect association between T1 fine motor skills and T2 early academic skills involving T2 executive function in high-altitude Tibetan preschoolers. EF may represent one statistical correlate involved in the association between early fine motor skills and subsequent early academic skills; however, because EF and early academic skills were assessed concurrently at T2, studies with temporally separated assessments are needed to evaluate the temporal ordering among these developmental processes. These observational findings may help inform hypotheses for future intervention research, but they do not demonstrate that training fine motor skills or executive function improves academic outcomes.

## Data Availability

The raw data supporting the conclusions of this article will be made available by the authors, without undue reservation.

## References

[B1] Adolph K. E. (2008). Learning to move. Curr. Dir. Psychol. Sci. 17, 213–218. doi: 10.1111/j.1467-8721.2008.00577.x 19305638 PMC2658759

[B2] Bao R. Wade L. Leahy A. A. Owen K. B. Hillman C. H. Jaakkola T. . (2024). Associations between motor competence and executive functions in children and adolescents: a systematic review and meta-analysis. Sports Med. 54, 2141–2156. doi: 10.1007/s40279-024-02040-1 38769244 PMC11329584

[B3] Cameron C. E. Brock L. L. Murrah W. M. Bell L. H. Worzalla S. L. Grissmer D. . (2012). Fine motor skills and executive function both contribute to kindergarten achievement. Child Dev. 83, 1229–1244. doi: 10.1111/j.1467-8624.2012.01768.x 22537276 PMC3399936

[B4] Carlson A. G. Rowe E. Curby T. W. (2013). Disentangling fine motor skills' relations to academic achievement: the relative contributions of visual-spatial integration and visual-motor coordination. J. Genet. Psychol. 174, 514–533. doi: 10.1080/00221325.2012.717122 24303571

[B5] Chen C. Stevenson H. W. (1988). Cross-linguistic differences in digit span of preschool children. J. Exp. Child Psychol. 46, 150–158. doi: 10.1016/0022-0965(88)90027-6

[B6] Cirino P. T. Miciak J. Ahmed Y. Barnes M. A. Taylor W. P. Gerst E. H. (2019). Executive function: association with multiple reading skills. Read. Writ. 32, 1819–1846. doi: 10.1007/s11145-018-9923-9 31680727 PMC6824553

[B7] Cole D. A. Maxwell S. E. (2003). Testing mediational models with longitudinal data: questions and tips in the use of structural equation modeling. J. Abnorm. Psychol. 112, 558–577. doi: 10.1037/0021-843X.112.4.558 14674869

[B8] Connor C. M. Day S. L. Phillips B. Sparapani N. Ingebrand S. W. McLean L. . (2016). Reciprocal effects of self-regulation, semantic knowledge, and reading comprehension in early elementary school. Child Dev. 87, 1813–1824. doi: 10.1111/cdev.12570 27264645 PMC5138137

[B9] Diamond A. (2000). Close interrelation of motor development and cognitive development and of the cerebellum and prefrontal cortex. Child Dev. 71, 44–56. doi: 10.1111/1467-8624.00117 10836557

[B10] Diamond A. (2013). Executive functions. Annu. Rev. Psychol. 64, 135–168. doi: 10.1146/annurev-psych-113011-143750 23020641 PMC4084861

[B11] Duncan G. J. Dowsett C. J. Claessens A. Magnuson K. Huston A. C. Klebanov P. . (2007). School readiness and later achievement. Dev. Psychol. 43, 1428–1446. doi: 10.1037/0012-1649.43.6.1428 18020822

[B12] Emslander V. Scherer R. (2022). The relation between executive functions and math intelligence in preschool children: a systematic review and meta-analysis. Psychol. Bull. 148, 337–369. doi: 10.1037/bul0000369

[B13] Friedman N. P. Miyake A. (2017). Unity and diversity of executive functions: individual differences as a window on cognitive structure. Cortex 86, 186–204. doi: 10.1016/j.cortex.2016.04.023 27251123 PMC5104682

[B14] Gashaj V. Oberer N. Mast F. W. Roebers C. M. (2019). The relation between executive functions, fine motor skills, and basic numerical skills and their relevance for later mathematics achievement. Early Educ. Dev. 30, 913–926. doi: 10.1080/10409289.2018.1539556

[B15] Gerstadt C. L. Hong Y. J. Diamond A. (1994). The relationship between cognition and action: performance of children 3 1/2–7 years old on a stroop-like day-night test. Cognition 53, 129–153. doi: 10.1016/0010-0277(94)90068-X 7805351

[B16] Ginsburg H. P. Baroody A. J. (2003). TEMA-3: Test of Early Mathematics Ability. Austin, TX: Pro-ed.

[B17] Gong Y. Dai X. (1988). China-Wechsler younger children scale of intelligence (C-WYCSI). Acta Psychol. Sin. 20, 30–42.

[B18] Goodway J. D. Ozmun J. C. Gallahue D. L. (2019). Understanding Motor Development: Infants, Children, Adolescents, Adults. Burlington, MA: Jones and Bartlett Learning.

[B19] Grissmer D. Grimm K. J. Aiyer S. M. Murrah W. M. Steele J. S. (2010). Fine motor skills and early comprehension of the world: two new school readiness indicators. Dev. Psychol. 46, 1008–1017. doi: 10.1037/a0020104 20822219

[B20] Hawes Z. Moss J. Caswell B. Seo J. Ansari D. (2019). Relations between numerical, spatial, and executive function skills and mathematics achievement: a latent-variable approach. Cogn. Psychol. 109, 68–90. doi: 10.1016/j.cogpsych.2018.12.002 30616227

[B21] Hill P. J. McNarry M. A. Mackintosh K. A. Murray M. A. Pesce C. Valentini N. C. . (2024). The influence of motor competence on broader aspects of health: a systematic review of the longitudinal associations between motor competence and cognitive and social-emotional outcomes. Sports Med. 54, 375–427. doi: 10.1007/s40279-023-01939-5 37989831 PMC10933160

[B22] Hogan A. M. Virues-Ortega J. Botti A. B. Bucks R. Holloway J. W. Rose-Zerilli M. J. . (2010). Development of aptitude at altitude. Dev. Sci. 13, 533–544. doi: 10.1111/j.1467-7687.2009.00909.x 20443973

[B23] Hongwanishkul D. Happaney K. R. Lee W. S. Zelazo P. D. (2005). Assessment of hot and cool executive function in young children: age-related changes and individual differences. Dev. Neuropsychol. 28, 617–644. doi: 10.1207/s15326942dn2802_4 16144430

[B24] Howard S. J. Melhuish E. (2017). An early years toolbox for assessing early executive function, language, self-regulation, and social development: validity, reliability, and preliminary norms. J. Psychoeduc. Assess. 35, 255–275. doi: 10.1177/0734282916633009 28503022 PMC5424850

[B25] Hua J. Gu G. Meng W. Wu Z. (2013). Age band 1 of the movement assessment battery for children-second edition: exploring its usefulness in mainland China. Res. Dev. Disabil. 34, 801–808. doi: 10.1016/j.ridd.2012.10.012 23220119

[B26] Karr J. E. Areshenkoff C. N. Rast P. Hofer S. M. Iverson G. L. Garcia-Barrera M. A. (2018). The unity and diversity of executive functions: a systematic review and re-analysis of latent variable studies. Psychol. Bull. 144, 1147–1185. doi: 10.1037/bul0000160 30080055 PMC6197939

[B27] Kim H. Duran C. A. K. Cameron C. E. Grissmer D. (2018). Developmental relations among motor and cognitive processes and mathematics skills. Child Dev. 89, 476–494. doi: 10.1111/cdev.12752 28181219 PMC5550379

[B28] Li Y. Wu X. Ye D. Zuo J. Liu L. (2024). Research progress on the relationship between fine motor skills and academic ability in children: a systematic review and meta-analysis. Front Sports Act Living 6:1386967. doi: 10.3389/fspor.2024.1386967 39850871 PMC11754413

[B29] Liu Y. Hong J. Yin X. Zhang F. Guo Y. Sun P. (2023). Relationship between cardiorespiratory fitness and executive function of Chinese Tibetan adolescents aged 13–18. J. Sci. Med. Sport 26, 610–615. doi: 10.1016/j.jsams.2023.09.003 37739853

[B30] Ltifi M. A. Cherni Y. Panaet E. A. Alexe C. I. Ben Saad H. Vulpe A. M. . (2025a). Mini-trampoline training enhances executive functions and motor skills in preschoolers. Children 12:1405. doi: 10.3390/children12101405 41153587 PMC12563777

[B31] Ltifi M. A. Meksi S. Alotaibi A. Chelly M. S. (2026a). Urban–rural differences in BMI status, 24-hour movement guideline compliance, executive functions, and motor skills in preschool children: a cross-sectional study. Front. Physiol. 17:1882530. doi: 10.3389/fphys.2026.1882530 42620496 PMC13485547

[B32] Ltifi M. A. Nejah K. Hammami F. Bîcă M. D. Zwierzchowska A. Wilk M. . (2026b). Associations between body mass index, movement behaviors, motor skills, inhibition and visuospatial working memory in preschool children: a cross-sectional study based on WHO references. Children 13:306. doi: 10.3390/children13020306 41749663 PMC12939077

[B33] Ltifi M. A. Turki O. Ben-Bouzaiene G. Pagaduan J. C. Okely A. Chelly M. S. (2025b). Exploring 24-hour movement behaviors in early years: findings from the SUNRISE pilot study in Tunisia. Pediatr. Exerc. Sci. 37, 94–101. doi: 10.1123/pes.2023-0152 38364818

[B34] Lu L. Liu H. (1998). The Peabody Picture Vocabulary Test-Revised in Chinese. Taipei: Psychological Publishing.

[B35] Marr D. Cermak S. Cohn E. S. Henderson A. (2003). Fine motor activities in head start and kindergarten classrooms. Am. J. Occup. Ther. 57, 550–557. doi: 10.5014/ajot.57.5.550 14527117

[B36] Maxwell S. E. Cole D. A. (2007). Bias in cross-sectional analyses of longitudinal mediation. Psychol. Methods 12, 23–44. doi: 10.1037/1082-989X.12.1.23 17402810

[B37] McClelland M. M. Cameron C. E. (2019). Developing together: the role of executive function and motor skills in children's early academic lives. Early Child. Res. Q. 46, 142–151. doi: 10.1016/j.ecresq.2018.03.014

[B38] McClelland M. M. Cameron C. E. Connor C. M. Farris C. L. Jewkes A. M. Morrison F. J. (2007). Links between behavioral regulation and preschoolers' literacy, vocabulary, and math skills. Dev. Psychol. 43, 947–959. doi: 10.1037/0012-1649.43.4.947 17605527

[B39] Meksi S. Ltifi M. A. Alfawaz W. Alotaibi A. Chelly M. S. (2026). Sex-specific BMI, 24-hour movement behaviors, motor skills, and executive functions in Tunisian preschool children: an observational cross-sectional study. Front. Physiol. 17:1853244. doi: 10.3389/fphys.2026.1853244 42245835 PMC13229776

[B40] Michel E. Molitor S. Schneider W. (2020). Executive functions and fine motor skills in kindergarten as predictors of arithmetic skills in elementary school. Dev. Neuropsychol. 45, 367–379. doi: 10.1080/87565641.2020.1821033 32942903

[B41] Miyake A. Friedman N. P. Emerson M. J. Witzki A. H. Howerter A. Wager T. D. (2000). The unity and diversity of executive functions and their contributions to complex “frontal lobe” tasks: a latent variable analysis. Cogn. Psychol. 41, 49–100. doi: 10.1006/cogp.1999.0734 10945922

[B42] Needham A. W. Nelson E. L. (2023). How babies use their hands to learn about objects: exploration, reach-to-grasp, manipulation, and tool use. WIREs Cogn. Sci. 14:e1661. doi: 10.1002/wcs.1661 37286193

[B43] Niermeyer S. Mollinedo P. A. Huicho L. (2009). Child health and living at high altitude. Arch. Dis. Child. 94, 806–811. doi: 10.1136/adc.2008.141838 19066173

[B44] Penaloza D. Arias-Stella J. (2007). The heart and pulmonary circulation at high altitudes. Circulation 115, 1132–1146. doi: 10.1161/CIRCULATIONAHA.106.624544 17339571

[B45] Peng P. Lin X. Ünal Z. E. Lee K. Namkung J. Chow J. . (2020). Examining the mutual relations between language and mathematics: a meta-analysis. Psychol. Bull. 146, 595–634. doi: 10.1037/bul0000231 32297751

[B46] Pesigan I. J. A. Cheung S. F. (2024). Monte Carlo confidence intervals for the indirect effect with missing data. Behav. Res. Methods 56, 1678–1696. doi: 10.3758/s13428-023-02114-4 37550469

[B47] Pitchford N. J. Papini C. Outhwaite L. A. Gulliford A. (2016). Fine motor skills predict maths ability better than they predict reading ability in the early primary school years. Front. Psychol. 7:783. doi: 10.3389/fpsyg.2016.00783 27303342 PMC4884738

[B48] Schmidt M. Egger F. Benzing V. Jäger K. Conzelmann A. Roebers C. M. . (2017). Disentangling the relationship between children's motor ability, executive function and academic achievement. PLoS One 12:e0182845. doi: 10.1371/journal.pone.0182845 28817625 PMC5560562

[B49] Shokrkon A. Nicoladis E. (2022). The directionality of the relationship between executive functions and language skills: a literature review. Front. Psychol. 13:848696. doi: 10.3389/fpsyg.2022.848696 35928417 PMC9343615

[B50] Spiegel J. A. Goodrich J. M. Morris B. M. Osborne C. M. Lonigan C. J. (2021). Relations between executive functions and academic outcomes in elementary school children: a meta-analysis. Psychol. Bull. 147, 329–351. doi: 10.1037/bul0000322 34166004 PMC8238326

[B51] Su R. Jia S. Zhang N. Wang Y. Li H. Zhang D. . (2024). The effects of long-term high-altitude exposure on cognition: a meta-analysis. Neurosci. Biobehav. Rev. 161:105682. doi: 10.1016/j.neubiorev.2024.105682 38642865

[B52] Thelen E. Schöner G. Scheier C. Smith L. B. (2001). The dynamics of embodiment: a field theory of infant perseverative reaching. Behav. Brain Sci. 24, 1–34. doi: 10.1017/S0140525X01003910 11515285

[B53] Turnbull K. L. P. DeCoster J. Downer J. T. Williford A. P. (2024). Elucidating Linkages of executive functioning to school readiness skill gains: the mediating role of behavioral engagement in the PreK classroom. Early Child. Res. Q. 69, 38–48. doi: 10.1016/j.ecresq.2024.07.001 39070245 PMC11271645

[B54] van Buuren S. Groothuis-Oudshoorn K. (2011). Mice: multivariate imputation by chained equations in R. J. Stat. Softw. 45, 1–67. doi: 10.18637/jss.v045.i03

[B55] Viterbori P. Usai M. C. Traverso L. De Franchis V. (2015). How preschool executive functioning predicts several aspects of math achievement in Grades 1 and 3: a longitudinal study. J. Exp. Child Psychol. 140, 38–55. doi: 10.1016/j.jecp.2015.06.014 26218333

[B56] Wiebe S. A. Espy K. A. Charak D. (2008). Using confirmatory factor analysis to understand executive control in preschool children: I. Latent structure. Dev. Psychol. 44, 575–587. doi: 10.1037/0012-1649.44.2.575 18331145

[B57] Williams K. (2019). Expressive Vocabulary Test, 3rd Edn. Minneapolis, MN: NCS Pearson.

[B58] Willoughby M. T. Blair C. B. (2016). Measuring executive function in early childhood: a case for formative measurement. Psychol. Assess. 28, 319–330. doi: 10.1037/pas0000152 26121388 PMC4695318

[B59] Willoughby M. T. Hudson K. (2023). Contributions of motor skill development and physical activity to the ontogeny of executive function skills in early childhood. Dev. Rev. 70:101102. doi: 10.1016/j.dr.2023.101102

[B60] Willoughby M. T. Magnus B. Vernon-Feagans L. Blair C. B. (2017). Developmental delays in executive function from 3 to 5 years of age predict kindergarten academic readiness. J. Learn. Disabil. 50, 359–372. doi: 10.1177/0022219415619754 26755570 PMC5266699

[B61] Wilson M. (2002). Six views of embodied cognition. Psychon. Bull. Rev. 9, 625–636. doi: 10.3758/BF03196322 12613670

[B62] Zhou Y. Tolmie A. (2024). Associations between gross and fine motor skills, physical activity, executive function, and academic achievement: longitudinal findings from the UK millennium cohort study. Brain Sci. 14:121. doi: 10.3390/brainsci14020121 38391696 PMC10887312

