# Supplementary material for: Fine motor skills at preschool age and later academic skills: an indirect-effects analysis involving executive function in high-altitude Tibetan preschoolers
Source: Front Psychol. 2026 Sep 8;17:1938612. doi: 10.3389/fpsyg.2026.1938612 (PMC13593777; doi:10.3389/fpsyg.2026.1938612)
Supplement: Supplementary file 1 [file Table_1.docx]

Supplementary Table S1. Descriptive comparison of participants retained and lost to follow-up on key T1 characteristics

| T1 characteristic | Retained at T2 (n = 198) | Lost to follow-up (n = 4) |
| --- | --- | --- |
| **Age, months** | 48.77 ± 6.82 | 45.91 ± 7.39 |
| **Fine motor skills** | 9.29 ± 2.75 | 7.75 ± 2.36 |
| **Sex, boys/girls** | 99/99 | 3/1 |

Note. Continuous variables are presented as mean ± SD. Because only four participants were lost to follow-up, the descriptive comparisons should be interpreted cautiously.

Supplementary Table S2. Variable-specific missingness before multiple imputation in the primary analytic cohort (N = 202)

| Variable | Missing, n | Missing, % |
| --- | --- | --- |
| **T1 measures** |  |  |
| Fine motor skills | 0 | 0.0 |
| Day–Night | 18 | 8.9 |
| Happy–Sad | 19 | 9.4 |
| Auditory working memory | 14 | 6.9 |
| Visual working memory | 14 | 6.9 |
| Cognitive flexibility | 13 | 6.4 |
| Mathematics | 61 | 30.2 |
| Receptive vocabulary | 41 | 20.3 |
| Expressive vocabulary | 55 | 27.2 |
| Nonverbal intelligence – Block Design | 48 | 23.8 |
| Nonverbal intelligence – Picture Completion | 47 | 23.3 |
| **T2 measures** |  |  |
| Day–Night | 4 | 2.0 |
| Happy–Sad | 4 | 2.0 |
| Auditory working memory | 4 | 2.0 |
| Visual working memory | 4 | 2.0 |
| Cognitive flexibility | 4 | 2.0 |
| Mathematics | 14 | 6.9 |
| Receptive vocabulary | 11 | 5.4 |
| Expressive vocabulary | 14 | 6.9 |
| Nonverbal intelligence – Block Design | 10 | 5.0 |
| Nonverbal intelligence – Picture Completion | 10 | 5.0 |
| Home language | 0 | 0.0 |
| Paternal education | 43 | 21.3 |
| Maternal education | 42 | 20.8 |
| Family annual income | 44 | 21.8 |

Note. Percentages were calculated using the primary analytic cohort (N = 202) as the denominator. Missingness is reported before multiple imputation. An observed T1 fine motor skills score was required for inclusion in the primary analytic cohort and was therefore fully observed within N = 202.
